# Construction and characterization of broad-host-range reporter plasmid suitable for on-line analysis of bacterial host responses related to recombinant protein production

**DOI:** 10.1186/s12934-019-1128-7

**Published:** 2019-05-07

**Authors:** Agnieszka Gawin, Karl Peebo, Sebastian Hans, Helga Ertesvåg, Marta Irla, Peter Neubauer, Trygve Brautaset

**Affiliations:** 1 Department of Biotechnology and Food Science, Norwegian University of Science and Technology, Sem Sælandsvei 6-8, 7491 Trondheim, Norway; 2 Center of Food and Fermentation Technologies, Akadeemia tee 15a, 12618 Tallinn, Estonia; 3 Bioprocess Engineering, Institute of Biotechnology, Technische Universität Berlin, Ackerstrasse 76, 13355 Berlin, Germany

**Keywords:** Fluorescent proteins, Recombinant protein production, Synthetic biology, Metabolic engineering, Microbial cell factory

## Abstract

**Background:**

Bacteria are widely used as hosts for recombinant protein production due to their rapid growth, simple media requirement and ability to produce high yields of correctly folded proteins. Overproduction of recombinant proteins may impose metabolic burden to host cells, triggering various stress responses, and the ability of the cells to cope with such stresses is an important factor affecting both cell growth and product yield.

**Results:**

Here, we present a versatile plasmid-based reporter system for efficient analysis of metabolic responses associated with availability of cellular resources utilized for recombinant protein production and host capacity to synthesize correctly folded proteins. The reporter plasmid is based on the broad-host range RK2 minimal replicon and harbors the strong and inducible XylS/*Pm* regulator/promoter system, the ppGpp-regulated ribosomal protein promoter P*rpsJ*, and the σ^32^-dependent synthetic tandem promoter P*ibpfxs*, each controlling expression of one distinguishable fluorescent protein. We characterized the responsiveness of all three reporters in *Escherichia coli* by quantitative fluorescence measurements in cell cultures cultivated under different growth and stress conditions. We also validated the broad-host range application potential of the reporter plasmid by using *Pseudomonas putida* and *Azotobacter vinelandii* as hosts.

**Conclusions:**

The plasmid-based reporter system can be used for analysis of the total inducible recombinant protein production, the translational capacity measured as transcription level of ribosomal protein genes and the heat shock-like response revealing aberrant protein folding in all studied Gram-negative bacterial strains.

**Electronic supplementary material:**

The online version of this article (10.1186/s12934-019-1128-7) contains supplementary material, which is available to authorized users.

## Background

Further advances in the field of recombinant protein production require continuous efforts concerning not only expression vector design but also rational genome engineering [1]. Typical host-related aspects affecting recombinant protein production are related to the availability of energy and protein synthesis components comprising amino acids, free ribosomal subunits and other translational factors. Limitations related to such host parameters are commonly observed as reduced cell growth, plasmid loss and poor production yields [1]. In many cases, though, it is difficult to predict and distinguish various host physiological effects, and there is a need for tools enabling high-throughput strain analysis in order to obtain more precise understanding of host-related responses related to overproduction of recombinant proteins [2, 3].

Depletion of amino acids is linked to the stringent stress response mediated by the guanosine tetraphosphate (ppGpp) alarmone, which among others down-regulates transcription of ribosomal protein (r-protein) and rRNA operons [4]. Although its mode of regulation may differ among microbial species [5], in Gram-negative bacteria, ppGpp primarily interacts with RNA polymerase (RNAP), in synergy with the RNAP-binding transcriptional factor DksA, to directly affect transcription [6, 7]; and the ppGpp binding site regions are well conserved in representatives of the Gammaproteobacteria [8]. In *Escherichia coli*, overproduction of recombinant proteins can lead to formation of inclusion bodies if there is an imbalance between the rates of protein synthesis and folding. Consequently, a σ^32^-related heat shock-like response is induced, which results in upregulation of the heat-shock proteins such as the chaperone DnaK [9–11]. Analysis of the *rpoH* genes encoding homologs of σ^32^ protein from the Alpha- and Gammaproteobacteria subgroups revealed sequence similarities that should reflect conserved function and regulation of σ^32^ in the heat-shock response [12]. These data are consistent with further observations that expression of σ^32^ homologs from *Serratia marcescens*, *Proteus mirabilis* and *Pseudomonas aeruginosa* in *E. coli* mutant strain lacking its own σ^32^ leads to the transcriptional activation of the heat-shock genes from the start sites normally used in *E. coli* [13].

A reporter system for monitoring the heat-shock like response to recombinant protein production has been reported previously [11], as a σ^32^-dependent tandem promoter, P*ibpfxs*, generated by fusion of *ibpAB* and *fxsA* promoters. The *ibpAB* operon encodes two small heat shock proteins involved in resistance to heat and superoxide stresses [14] and FxsA is an inner membrane protein whose expression is reported to be strongly associated with accumulation of misfolded proteins in the cells [15]. The P*ibpfxs* tandem promoter is characterised by a low basis level and strong transcriptional activation by the accumulation of aggregation prone proteins in inclusion bodies [11, 16].

Also, a fluorescence-based reporter system for the analysis of ribosome assembly was previously created and extensively characterized [17] and further used to measure the ribosome dynamics in *E. coli* [18].

**Fig. 1:**
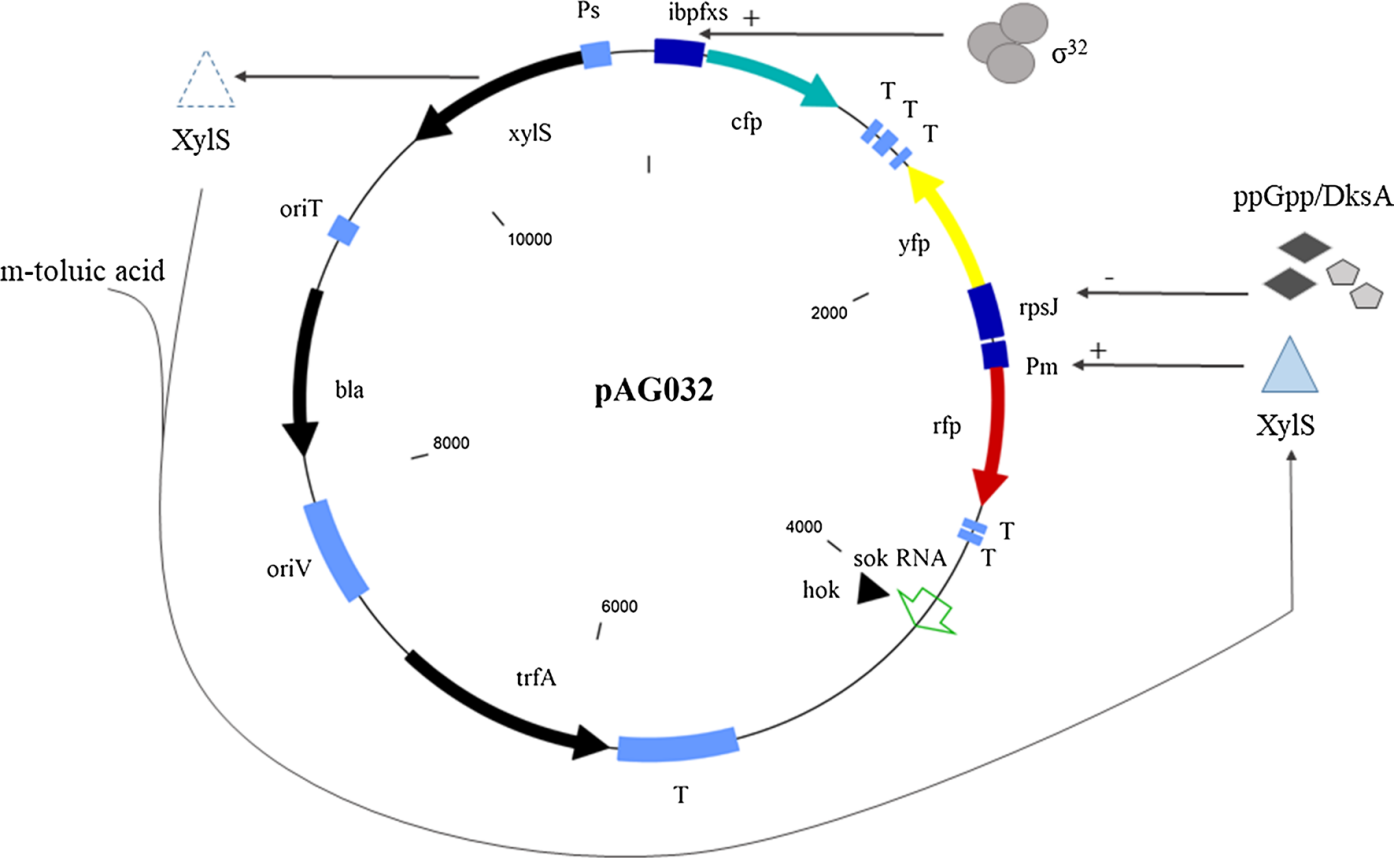
Genetic map of the pAG032 reporter plasmid (11,238 nt large) including representation of the control mechanisms of the three promoter/reporter pairs. The XylS/*Pm* regulator/promoter is inducible by benzoic acid derivatives such as m-toluic acid, while the P*rpsJ* and P*ibpfxs* promoters are responsive to intracellular concentrations of ppGpp and σ^32^, respectively. *oriT*, origin for conjugative transfer; *bla*, gene conferring ampicillin resistance; *oriV*, origin of vegetative replication; *trfA*; gene encoding an essential initiator protein TrfA; T, transcription terminators; *hok*/*sok* RNA, *hok*-*sok* suicide elements; *Ps*, σ^70^-dependent constitutive promoter; *xylS*, gene encoding the positive transcriptional regulator XylS

Here, we have taken these previous approaches one step further and constructed a versatile plasmid-based reporter system designated pAG032 (Fig. 1) based on the broad-host range replicon RK2 [19]. The plasmid contains distinct promoters controlling synthesis of three spectrally separable fluorescent proteins mCherry (RFP), mVenus (YFP), and mCerulean (CFP). Cox et al. [20] previously described plasmid pZS2-123 harbouring three inducible promoters expressing these fluorescent proteins and they showed how these reporters can be quantified to independently monitor genetic regulation and noise in single cells of *E. coli*. In our study, we used the inducible XylS/*Pm* regulator/promoter system originating from *Pseudomonas putida* TOL plasmid pWW0 to control production of a model protein, RFP. It was proven that this system is suitable for tightly regulated and high-level heterologous protein production in *E. coli* and other Gram-negative bacteria [21]. Next, to monitor for variations in ribosome synthesis in host cells, P*rpsJ* promoter of the *rpsJ* ribosomal protein gene was chosen to control *yfp* expression. In *E*. *coli*, P*rpsJ* coordinates transcription of the S10 operon encoding the 30S subunit of the ribosome. The P*rpsJ* belongs to r-protein promoters that are strongly inhibited by ppGpp and DksA transcriptional factors in the cells [4, 22]. The transcriptional regulation of *rpsJ* gene plays a major role when nutrient concentrations are insufficient to sustain the requirements for cell growth due to the enhanced precursor and energy demand required for biomass synthesis [23, 24]. Finally, the *cfp* reporter gene was placed under control of P*ibpfxs* to enable monitoring of the heat shock-like responses.

We characterized applicability of the constructed reporter plasmid as a novel tool to analyse host responses relevant for recombinant protein production in *E. coli*. Moreover, the broad-host range properties of pAG032 were confirmed by performing additional analyses in two other biotechnologically relevant Gammaproteobacteria, *Pseudomonas putida* and *Azotobacter vinelandii*.

## Results

### Design and construction of the broad-host-range reporter plasmid pAG032

As described above, the plasmid system was designed to analyze metabolic responses associated with availability of cellular resources utilized for recombinant protein production. We constructed the system (Fig. 1) by placing a gene encoding RFP under control of the *Pm* promoter positively regulated by XylS transcriptional factor. XylS/*Pm* has been widely used for regulated and high-level recombinant protein production in *E. coli* and other Gram-negative bacteria [21], and among all functional elements of the reporter plasmid RFP then serves as an indicator of the target protein production. In addition to inducible XylS/*Pm*, we used two stress-responsive promoters, P*rpsJ* and P*ibpfxs*. The expression of *yfp* is driven by the P*rpsJ*, which is significantly upregulated in response to optimal nutrient conditions and deficiencies of ribosomal components. Finally, expression of *cfp* relies upon the σ^32^-dependent P*ibpfxs* promoter [11] responding to heat shock and misfolded proteins. Transcriptional terminators were placed downstream of each reporter gene to avoid problems related to transcriptional read-through [20]. The pAG032 plasmid is based on the broad-host-range RK2 minimal replicon thereby ensuring that the plasmid is applicable in many different Gram-negative bacterial species [19]. The minimal RK2 replicon consists of the origin of vegetative replication (*oriV*) and its cognate *trfA* gene encoding TrfA replication initiator protein [19, 25]. The copy number of the plasmid is 5–7 per genome, and this number can easily be changed to different numbers up to above 100 copies per genome by replacing *trfA* with available mutant versions of *trfA* gene [26]. The latter option could be valuable to monitor metabolic burden and other responses associated with increased recombinant protein production and increased plasmid copy numbers. The pAG032 further contains *oriT* enabling conjugative transfer and the *hok*-*sok* suicide elements previously demonstrated to ensure segregational stability of RK2-based plasmid also under high-cell density cultivations [27].

Construction of pAG032 was done based on the expression plasmid pVB-1E0B1 IL-1RA (Table 1) in a step-wise process involving creation of versions containing one (pAG002, pAG014, and pAG017) and two (pAG019, pAG024 and pAG028) reporter genes used in the final reporter plasmid pAG032. In all cases, the functionality of the plasmid intermediates was tested for production of the relevant active reporter proteins in *E. coli* (data not shown). Next, we tested the functionality of pAG032 by individually monitoring expression level of the three reporter proteins in recombinant *E. coli* BW25113 (pAG032) cells cultivated under different conditions causing various stress responses as relevant for the particular promoter/reporter pair being investigated.

**Table 1:** Plasmids used in this study

Name	Key features	Source
pZS2-123	*cfp* under control of P_LtetO-1_, *yfp* under control of P_LlacO-1_, *rfp* under control of P_lac/ara-1_, Kan^r^	[20]
pibpfxsT7lucA	*ibpfxs*::*lucA* reporter unit, Amp^r^	[11]
pJB658	RK2 replicon, *trfA* gene, XylS/*Pm* regulator/promoter system, Amp^r^	[25]
pVB-1E0B1 IL-1RA	RK2 replicon, *trfA* gene with *cop*-*271* mutation, XylS/*Pm* regulator/promoter system, *hok*-*sok* suicide system, Amp^r^	Vectron Biosolutions
pAG001	RK2 replicon, *trfA* gene, *hok*-*sok* suicide system, Amp^r^	This study
pAG002	pAG001 plasmid with *rfp* under control of XylS/*Pm*, Amp^r^	This study
pAG014	pAG001 plasmid with *cfp* under control of *ibpfxs*, Amp^r^	This study
pAG017	pAG001 plasmid with *yfp* under control of *rpsJ*, Amp^r^	This study
pAG019	pAG001 plasmid with *rfp* under control of XylS/*Pm* and *yfp* under control of *rpsJ*, Amp^r^	This study
pAG024	pAG001 plasmid with *rfp* under control of XylS/*Pm* and *cfp* under control of *ibpfxs*, Amp^r^	This study
pAG028	pAG001 plasmid with *cfp* under control of *ibpfxs* and *yfp* under control of *rpsJ*, Amp^r^	This study
pAG032	pAG001 plasmid with *rfp* under control of XylS/*Pm*, *cfp* under control of *ibpfxs* and *yfp* under control of *rpsJ*, Amp^r^	This study

### Changes of *yfp* expression controlled by P*rpsJ* promoter in *E. coli* cells exposed to sub-lethal concentrations of translation-inhibiting antibiotic chloramphenicol

**Fig. 2:**
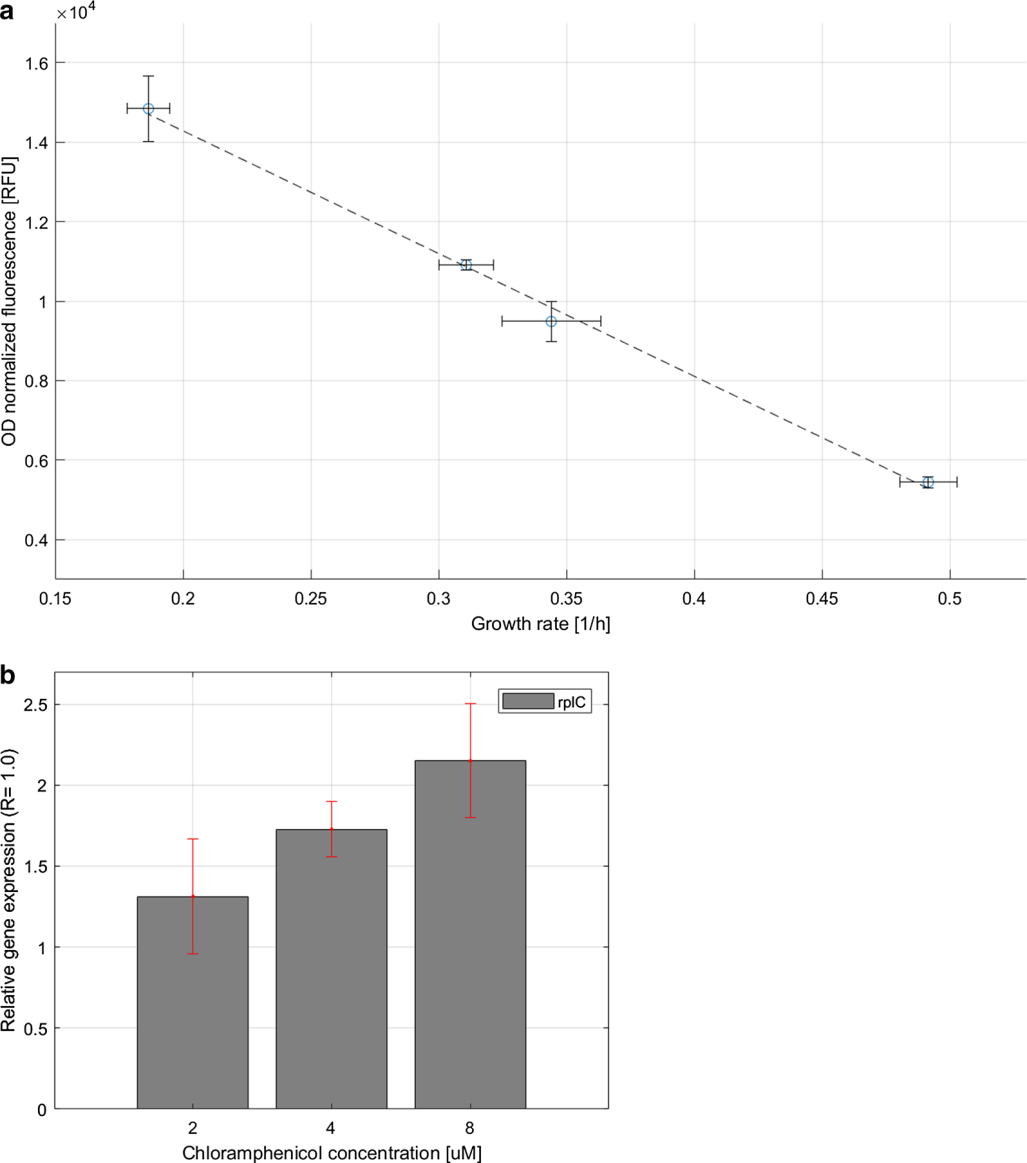
Transcriptional upregulation of P*rpsJ* in response to cell growth in various sub-lethal doses of chloramphenicol, measured as a correlation of P*rpsJ*-dependent expression of YFP activity and growth rate (**a**) or *rplC* transcript accumulation (**b**). The growth rates were calculated based on change in OD_600nm_ values measured every 1 h over a period of 3 h after adding chloramphenicol. OD_600nm_ normalized fluorescence values were calculated from measurements taken 3 h after adding chloramphenicol. RNA samples collection was performed after 15-min incubation of the recombinant cells in the presence of chloramphenicol. Relative gene expression (ΔΔCt) is presented as the level of *rplC* transcript in samples treated with chloramphenicol (2, 4, 8 μM) relative to its abundance in reference samples (0 μM). The data presented are from three independent biological replica (average ± SD)

The translational capacity is one of limiting factors for recombinant protein production rate [1] and bacterial growth rate [28]. Chloramphenicol is a translation-inhibiting antibiotic and it has previously been demonstrated that exposure of *E. coli* cells to sub-lethal chloramphenicol concentrations is accompanied by transcriptional upregulation of rRNA and ribosomal protein genes as a response to slow translation and accumulation of amino acid precursors. The result is an elevated intracellular RNA/protein ratio [29]. To test the responsiveness of the P*rpsJ* promoter to such conditions, recombinant *E. coli* cells were cultivated using different sub-lethal concentrations of chloramphenicol and assayed for YFP fluorescence intensity. The results showed that increased concentrations of chloramphenicol in the growth medium led to the expected reduced growth rate of the cells, and also, to the increased YFP fluorescence levels in comparison to control cells, indicating transcriptional upregulation of P*rpsJ* (Fig. 2a). The elevated YFP fluorescence level was inversely proportional to the growth rate of the cells, and supplementation of growth media with antibiotic concentrations of 2, 4 and 8 μM resulted in 1.7-, 2.0- and 2.7-fold increased YFP activity, respectively, in comparison to the cells cultivated in the absence of chloramphenicol.

In order to confirm that chloramphenicol treatment affects transcriptional upregulation of ribosomal protein genes, we measured transcription levels of the chromosomal *rplC* gene, which is part of the *rpsJ* (S10) operon in *E. coli* and encodes the 50S ribosomal subunit protein L3. The *rplC* transcript level was measured by quantitative PCR in RNA samples extracted from untreated *E. coli* cells and cells cultivated for 15 min after addition of increasing doses (2, 4 and 8 μM) of chloramphenicol [30]. The results showed that *rplC* transcript levels increased with increasing concentrations of chloramphenicol (Fig. 2b), in agreement with the YFP activity results (Fig. 2a).

### Elevated expression of *cfp* from P*ibpfxs* in cells exposed to azetidine is likely caused by protein misfolding and aggregation

**Fig. 3:**
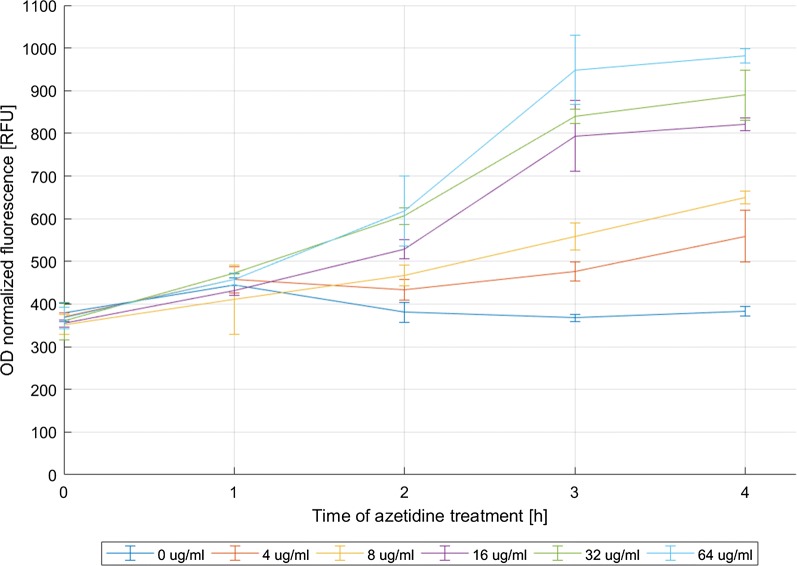
Time-course-activity measurements of YFP expressed from P*ibpfxs* in *E. coli* BW25113 (pAG032) cultivated in the presence of different concentrations (0, 4, 8, 16, 32 and 64 μg/ml) of azetidine. Incorporation of azetidine during protein synthesis promotes formation of abnormal proteins that tend to misfold and aggregate. The OD_600nm_ normalized fluorescence values were calculated based on the measurements taken every 1 h up to 4 h after addition of azetidine. The data presented are from three independent biological replicas (average ± SD)

Recombinant overexpression sometimes results in protein misfolding and aggregation. Previously, it has been shown [31–33] that incorporation of amino acid analogues during protein synthesis leads to formation of proteins with abnormal conformation and, prior to proteolytic degradation, such proteins form insoluble aggregates. This causes transcriptional upregulation of various heat shock chaperones regulated by the sigma factor σ^32^. In the plasmid pAG032, the σ^32^ dependent P*ibpfxs* tandem promoter controls *cfp* expression. To test if this promoter/reporter pair can be used to monitor cellular response to protein misfolding and aggregation, we cultivated recombinant *E. coli* cells in the presence of increasing concentrations (0, 4, 8, 16, 32 and 64 μg/ml) of a proline analogue—azetidine. Samples were collected at various time points after azetidine addition (1, 2, 3 and 4 h) and analysed for CFP fluorescence, and the results are presented in Fig. 3. As anticipated, CFP fluorescence intensity increased with increasing time and dose of azetidine. The incubation with 4, 8, 16, 32 and 64 μg/ml azetidine for 4 h resulted in 1.5-, 1.7-, 2.1-, 2.3- and 2.6-fold increase in CFP fluorescence, respectively, compared to the untreated cells.

### Simultaneous fluorescence measurement of reporter proteins YFP and CFP in cells cultivated in different growth media indicates host effects related to ribosome synthesis rates and σ^32^-mediated responses

**Fig. 4:**
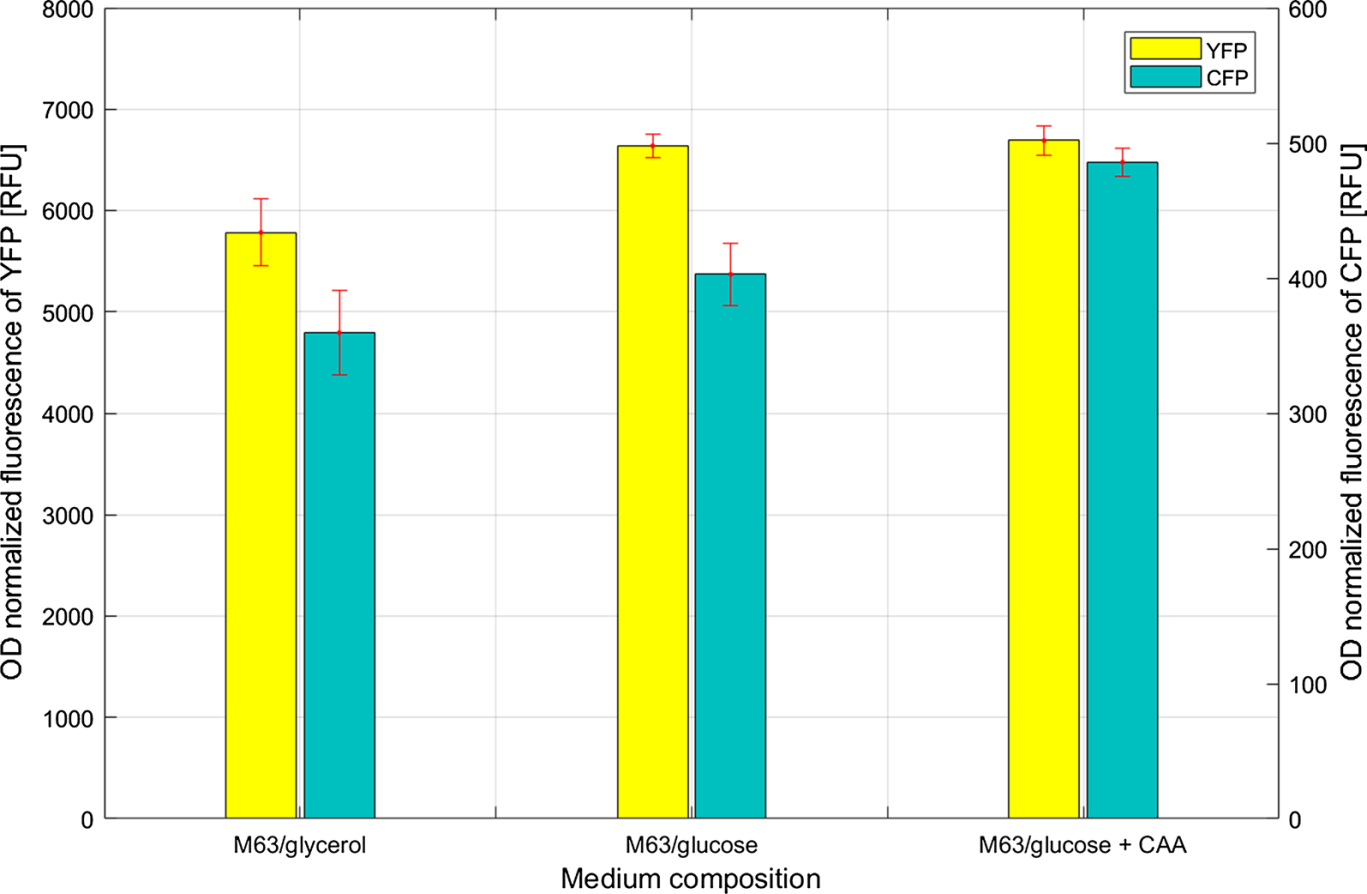
Recombinant cells were cultivated in minimal medium supplemented with glycerol (M63/glycerol), glucose (M63/glucose) and glucose + casamino acids (M63/glucose + CAA), to ensure different growth rates (Additional file 1: Table S1). The OD_600nm_ normalized fluorescence values were calculated based on measurements taken 3 h after a time point when all the cultures displayed approximate OD_600nm_ (~ 0.35). The data presented are from three independent biological replicas (average ± SD)

The important property of RFP, CFP and YFP as reporter proteins is that they have been reported to exhibit distinct fluorescence signals [15] allowing simultaneous and independent monitoring of each reporter protein fluorescence with regard to the studied parameters. We took advantage of that and studied YFP and CFP fluorescence in recombinant cells cultivated in three different growth conditions; M63 minimal medium supplemented with glycerol (M63/glycerol), glucose (M63/glucose), or glucose with casamino acids (M63/glucose + CAA). These growth media were chosen to promote different growth rates that should affect expression level of YFP from the P*rpsJ* promoter. As presented in Fig. 4, the fluorescence of the two reporter proteins was individually measured and quantified. The results show that the fluorescence of both proteins increased slightly (up to 1.2-fold) as a consequence of use of media stimulating higher cell growth rates. The increase was approximately proportional to that of the growth rate (Additional file 1: Table S1). The YFP data are in agreement with previous reports indicating that fast growing bacteria are expected to have higher demand for ribosomal proteins [28]. In addition, it was previously suggested that fast growth promotes increased rates of cellular protein synthesis, which may become a burden to the folding machinery [34, 35], and in that case increased *cfp* expression from P*ibpfxs* would be expected which is in accordance with results shown in Fig. 4.

### Inducible expression of *rfp* from XylS/*Pm* varies under different growth conditions

**Fig. 5:**
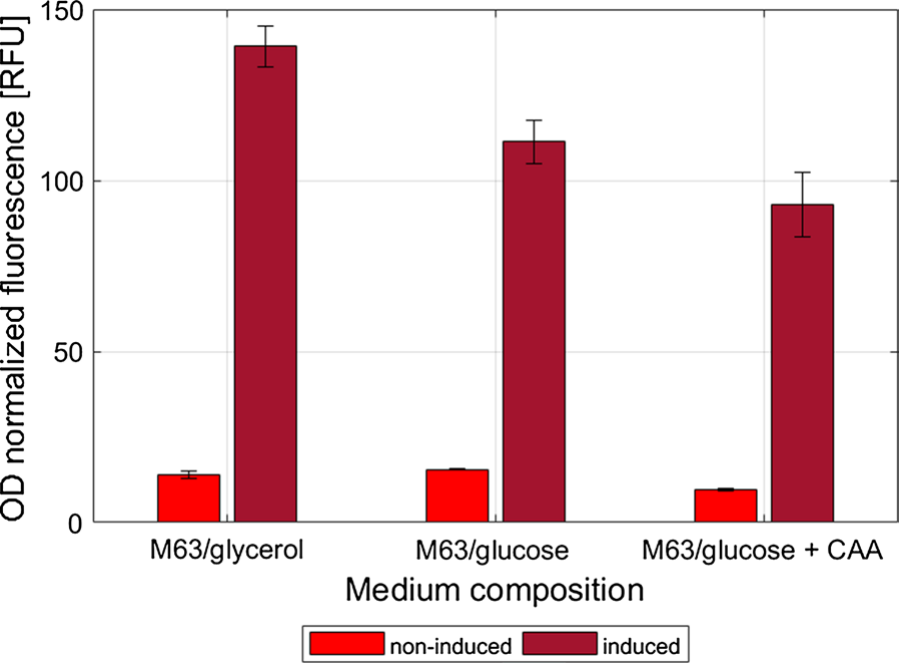
Responsiveness of the XylS/*Pm* reporter unit during cultivation of *E. coli* BW25113 in M63 medium supplemented with either glycerol (M63/glycerol), glucose (M63/glucose) or glucose and casamino acids (M63/glucose + CAA). The OD_600nm_ normalized fluorescence values were calculated from measurements taken at the time point corresponding to 3 h after the induction. The OD_600nm_ of the samples at the point of induction was similar for all cultures despite differences in the growth rate. The data presented are from three independent biological replica (average ± SD)

The XylS/*Pm* expression cassette has been widely used for high-level recombinant protein production in *E. coli* and other Gram-negative bacteria, and its tight regulation has been demonstrated to be favorable when expressing cell-toxic heterologous proteins under high-cell density conditions [27, 36]. In this study, the reporter gene *rfp* was put under control of the XylS/*Pm* in order to characterize this expression system in the context of plasmid pAG032. Recombinant cells were cultivated in three different growth media M63/glycerol, M63/glucose, and M63/glucose + CAA, as described above. Expression of *rfp* was induced by addition of m-toluic acid (1 mM), and the results are shown in Fig. 5. The *rfp* expression was highly inducible and levels of RFP fluorescence of induced cultures decreased with increasing cell growth rates of the cells. The highest RFP fluorescence was observed in cells growing on M63/glycerol, which was 1.5-fold higher than in case of the fastest-growing cells cultivated on M63/glucose + CAA. Still, it should be noted that the growth rate after induction with m-toluate was most strongly inhibited for the cells growing in M63/glucose + CAA (Additional file 1: Table S1).

### Plasmid pAG032 functions in other Gammaproteobacteria, *Pseudomonas putida* and *Azotobacter vinelandii*, indicating its potential as a broad-host-range tool

The broad-host range properties of the XylS/*Pm* system have been well documented, and importantly the inducer, m-toluate, enters cells via passive diffusion and most bacteria cannot metabolize it [21]. The P*rpsJ* and P*ibpfxs* promoters of pAG032 as well as the reporter genes used are not well studied in species other than *E. coli*. Therefore pAG032 was transformed to *Pseudomonas putida* KT2440 (TOL plasmid cured strain) and to *Azotobacter vinelandii* OP (UW) to explore its broad-host range potential. These two organisms were selected as relevant model hosts due their biotechnological applications for production of recombinant proteins and biopolymers [37, 38].

**Fig. 6:**
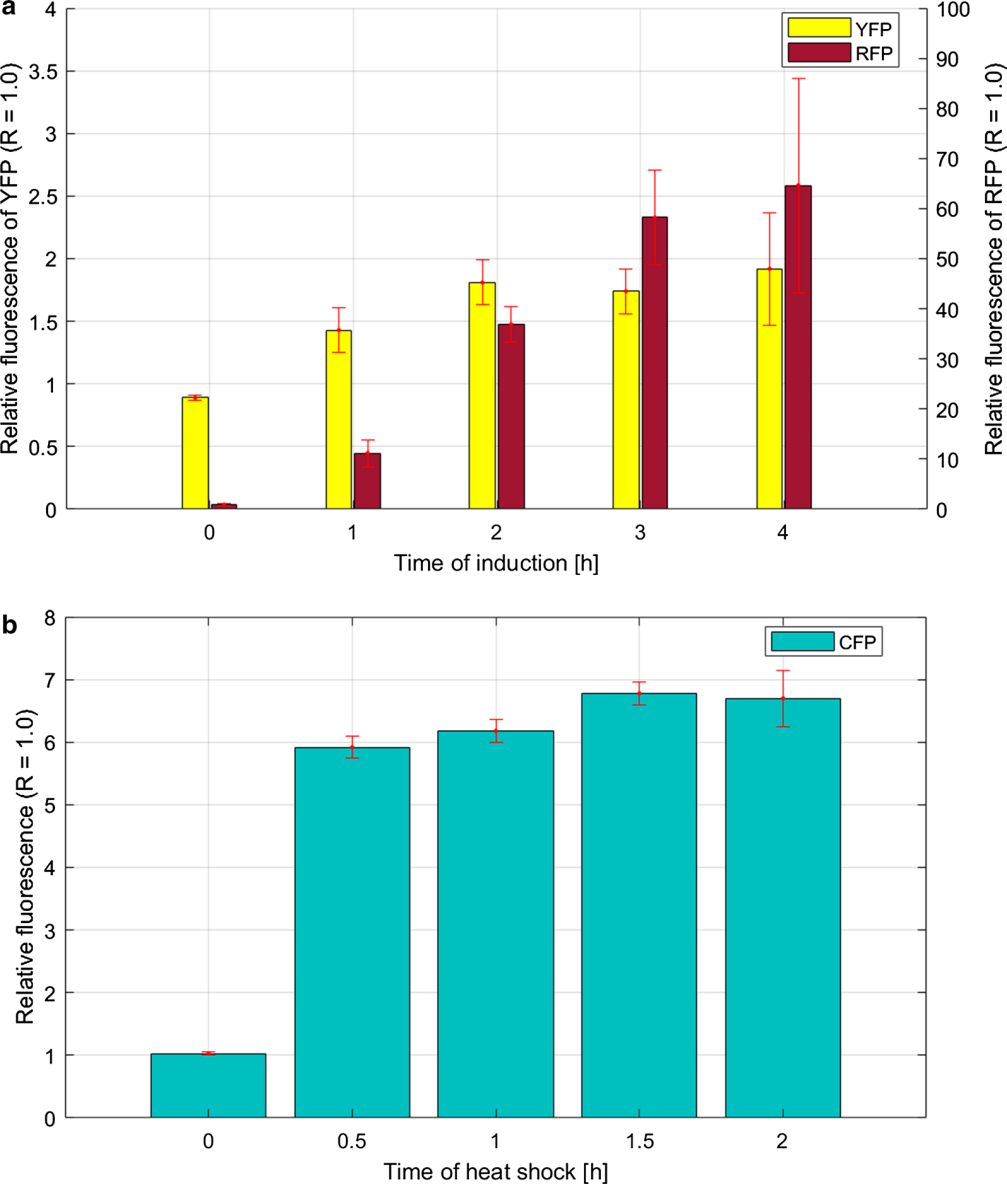
Responsiveness of the P*rpsJ* and XylS/*Pm* reporter units during cultivation of *P. putida* KT2440 under induced conditions (**a**) and the course of the P*ibpfxs* activity during cultivation at 42 °C (**b**). Data represent relative fluorescence levels shown as a ratio of OD_600nm_ normalized fluorescence values at different time points under conditions of induction or heat shock to the OD_600nm_ normalized fluorescence intensity of the reference cultures (R) growing in the absence of the inducer at 30 °C. The OD_600nm_ normalized fluorescence values were calculated based on measurements taken every 1 h up to 4 h after the induction and every 0.5 h up to 2 h after the temperature shift. The data presented are from three independent biological replica (average ± SD)

First, we analysed expression of all three reporter proteins in recombinant *P*. *putida* cells in the presence and absence of m-toluic acid (1 mM) under standard growth conditions (see “Methods” section), and the results are presented in Fig. 6a. As expected, *rfp* expression from the XylS/*Pm* was tightly regulated, and the induction ratio was found to be 64-fold 4 h after the induction. Interestingly, the expression level of *yfp* from ribosomal promoter P*rpsJ* was 1.9-fold upregulated upon 4-h incubation in the presence of m-toluic acid. *Cfp* expression from P*ibpfxs* remained constant irrespective of the presence of m-toluic acid (data not shown). To test for specific responsiveness of the latter promoter in *P. putida,* we performed an additional experiment where the cell cultures were shifted from 30 °C to 42 °C to induce a heat shock response. It was previously suggested that *P*. *putida* employs principally the same system as that in *E. coli* to control the activity and quantity of σ^32^ in response to heat stress and protein misfolding and aggregation [39]. The results of our experiment (Fig. 6b) showed that *cfp* expression from P*ibpfxs* increased 5.9-fold already 30 min after the temperature shift, in comparison to the control cells cultivated at 30 °C.

**Fig. 7:**
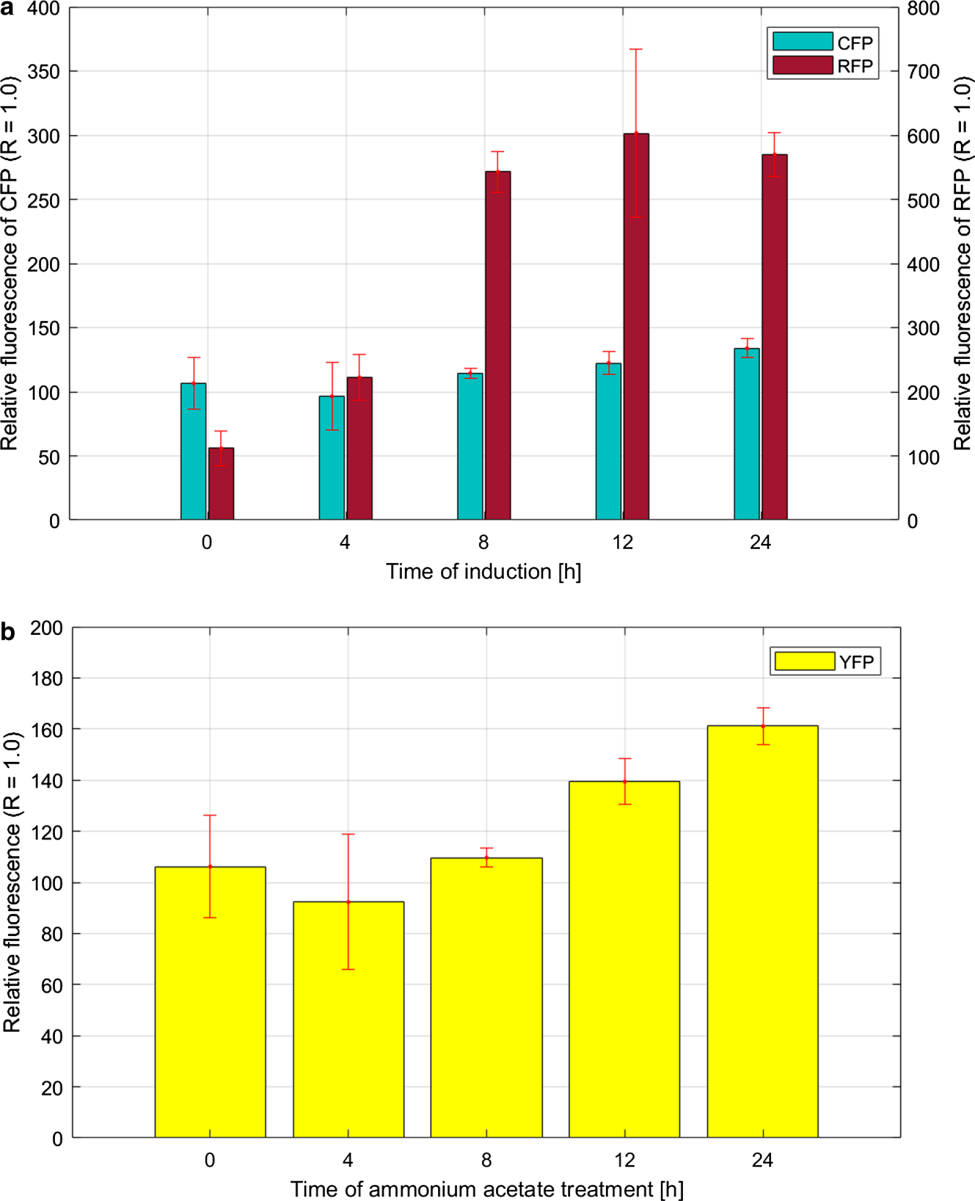
Responsiveness of the P*ibpfxs* and XylS/*Pm* reporter units during cultivation of *A. vinelandii* OP (UW) under induced conditions (**a**) and the activity of P*rpsJ* ribosomal promoter after the addition of ammonium acetate (**b**). Data represent relative fluorescence levels expressed as a ratio of OD_600nm_ normalized fluorescence values at different time points under conditions of induction or ammonium acetate supplementation to the OD_600nm_ normalized fluorescence intensity of the reference cultures (R) growing in the absence of the inducer or ammonium acetate. The OD_600nm_ normalized fluorescence values were calculated based on measurements taken up to 24 h after the induction or ammonium acetate supplementation. The data presented are from technical triplicates (average ± SD)

Next, we performed analogous studies by cultivating the recombinant *A*. *vinelandii* cells with and without m-toluic acid (0.5 mM), and the results showed that *rfp* expression from the XylS/*Pm* increased up to 6-fold after 12-h growth in the presence of the inducer (Fig. 7a). The induction window of the XylS/*Pm* for this host was lower than in case of *E. coli* and *P. putida* (see above). We noticed that the *cfp* expression level from the σ^32^-dependent P*ibpfxs* promoter increased slightly; up to 1.34-fold after 24 h growth in the presence of m-toluic acid compared to reference cells. The expression level of *yfp* from the P*rpsJ* promoter did not change significantly upon addiction with m-toluate (data not shown). As *A. vinelandii* is a nitrogen-fixing bacterium, it is therefore routinely cultivated in media lacking any nitrogen source. Nitrogen fixation is energy-demanding process, and both the nitrogen fixation and the partially uncoupled respiration protecting the nitrogenase against oxygen result in large demand for carbon that cannot be utilized for growth [37]. Therefore, we decided to test if supplementation with 10 mM ammonium acetate used as the nitrogen source, affects growth rate and expression of *yfp* from the P*rpsJ* promoter similarly to the effect observed for *E. coli* cultivated in richer media (Fig. 4). As shown in Fig. 7b, up to 1.6-fold increased YFP fluorescence was observed after 24 h of incubation in the presence of ammonium acetate in comparison to control cultures.

## Discussion

The advent of efficient and precise genome engineering tools such as CRISPR/Cas9 significantly accelerated the traditional endonuclease- and recombineering-based strain development processes useful for metabolic engineering [40, 41]. According to Mahalik et al. [1] engineering of bacterial hosts for improved capability for recombinant protein production requires removing translational bottlenecks and redirecting the metabolic flux away from biomass formation towards target protein synthesis. Moreover, the engineered cells should be characterized for their ability to efficiently fold and export proteins in order to prevent aggregation of the newly synthesized proteins inside the cytoplasm. In this work, we created the reporter system pAG032 that allows monitoring of translational capacity of bacterial cells and stresses related to protein misfolding. Our results confirmed that the activity of the P*rpsJ* ribosomal promoter of pAG032 corresponded to transcriptional upregulation of the chromosomal ribosome synthesis gene *rplC* in *E. coli*. Furthermore, by combining the P*rpsJ* ribosomal promoter reporter with the well-characterized σ^32^-dependent P*ibpfxs* on pAG032, we enabled monitoring of heat shock-like stress responses that may result from genome engineering aiming solely for improved translational capacity. The responsiveness of the P*ibpfxs* promoter controlling expression of *cfp* reporter gene was confirmed in our study by analysis of CFP fluorescence in cell cultures cultivated in a presence of the proline analogue azetidine. The activity of these two stress-responsive reporter units was further quantified by using different growth conditions of the recombinant *E. coli* cells. As P*rpsJ*-dependent YFP fluorescence intensity was monitored during the exponential growth phase with nutrient concentrations sufficient for steady growth rate [10, 18], the activity of this ribosomal reporter unit increased in correspondence to the increased growth rate. These results are in agreement with previously reported data indicating a positive correlation of ribosomal protein synthesis with growth rate in *E*. *coli* [42]. Our results using *A. vinelandii* as an alternative host also showed the expected growth-related expression of YFP from P*rpsJ*. Moreover, it has previously been proposed that high bacterial growth rate promotes increased rates of cellular protein synthesis, which may become a burden to the protein folding machinery [34, 35]. This may explain why the expression of *cfp* from σ^32^-dependent P*ibpfxs* promoter also increased in response to elevated growth rate of the *E. coli* cells.

The third reporter unit of pAG032 is the inducible XylS/*Pm* system controlling expression of RFP, and we chose its non-mutated version previously shown to display very low background expression to ensure tightly regulated and high-level induced expression of, in principle, any target protein using pAG032 [43]. Contrary to YFP and CFP, expression level of RFP from the XylS/*Pm* promoter decreased with the increasing growth rate. According to a model proposed by Klumpp et al. [44], the concentrations of constitutively expressed regulators is reduced with increased cell growth rate, mainly because of the increase of cell volume. It was plausible to assume that such effects can explain our results with RFP.

We constructed the reporter system to monitor product formation under different growth conditions by using the XylS/*Pm* promoter system, and we demonstrated that this expression cassette can be used in combination with the two other stress-responsive reporter units. As we see it, expression of the individual reporter genes used in this study is likely not independent. For example, a dependence is indicated by the fact the σ^32^-mediated heat shock response may induce production of ppGpp [45, 46] leading to the P*rpsJ* inhibition or by influence of inducer on upregulation of stress-responsive promoters (Additional file 1: Figure S1). Still, even under the assumption that recombinant expression of YFP and CFP may impose some stress to the host cells, in addition to the stress caused by inducible expression from XylS/*Pm*, the reporter system can discriminate between the effects dependent on different strain background during comparative studies. Such analysis of variation in expression of three interdependent reporter units in a subset of different genetic variants of *E*. *coli* has been previously demonstrated by Cardinale et al. [47].

One important aim was to design and construct pAG032 with promoters and a replicon suitable for broad-host range applications, and we were able to demonstrate its usefulness and detect activity and functionality of all promoters and concomitant reporter proteins in *P*. *putida* and *A*. *vinelandii* both representing the Gammaproteobacteria subgroup. The detected expression levels of the reporter proteins in *A. vinelandii* were low compared to those obtained in *E. coli* and *P. putida*, possibly due to non-optimal codons of reporter genes in this host. Moreover, plasmid derivative pAG028, containing the two promoter/reporter cassettes P*rpsJ/yfp* and P*ibpfxs/cfp*, can also possibly prove to be useful for analysing host effects in combination with overproduction of any target protein of interest expressed from a separate compatible expression vector and in any Gram-negative species.

## Conclusions

In this study, we created a novel reporter system suitable for monitoring of native stress responses and translational capacity in *E. coli* as well as in other Gram-negative bacteria. The system is based on individual expression of three different fluorescent proteins from three different promoters, which makes it useful for high-throughput screening accompanying effective strain development studies and for on-line monitoring of host parameters during cultivation. Characterization of cells in terms of the stringent and heat shock-like stress responses should allow for selection of strains displaying improved translational capacity and ability to synthesise correctly folded proteins.

## Methods

### Strains, media and growth conditions

*Escherichia coli* K-12 BW25113 [48] served as an expression host for studying the responsiveness of the reporter system. *E. coli* DH5α was used for cloning purposes. *P. putida* KT2440 (TOL plasmid cured derivative [49]) and *A. vinelandii* OP (UW) [37] were utilized as alternative host organisms. *E. coli* and *P. putida* were routinely grown at 37 °C or 30 °C, respectively, with 225 rpm shaking in liquid LB medium or on solid LB plates, unless stated otherwise. The M63 medium used in this study was composed as described by others [50] and supplemented with 0.2% (w/v) glycerol or 0.2% (w/v) glucose and/or 0.1% (w/v) Casamino acids. *A. vinelandii* was routinely grown in liquid Burk’s medium (pH 7.2) at 30 °C, 225 rpm shaking [51]. For plasmid selection in *E. coli*, *P. putida* and *A. vinelandii*, ampicillin was used at concentrations 100 μg/ml, 500 μg/ml and 25 μg/ml, respectively.

### Plasmids construction

Plasmids used as templates or constructs that were generated in this study are listed in Table 1. DNA sequences of the PCR primers used during cloning steps described below are presented in Table 2. Plasmid pAG001 was used as a starting point for the construction of various intermediate versions of the reporter system. For the construction of pAG001, pJB658 and pVB-1E0B1 IL-1RA were digested with EcoRI and ApaLI. The fragment corresponding to the pVB-1E0B1 IL-1RA backbone and the *trfA* gene of pJB658 were ligated. The resulting pAG001 was further digested with NdeI and NotI. In order to construct pAG002, the *rfp* region together with T7TE+ terminator was amplified from pZS2-123 using RfpF_NdeI and RfpR_NotI and the PCR-fragment was digested with NdeI and NotI prior to insertion into pAG001. Plasmid pAG014 was created by Gibson assembly [52] of three fragments: NotI/MfeI-digested pAG001 backbone, the region comprising *cfp* gene and RNAI terminator amplified from pZS2-123 using 014_cfp_F and 014_cfp_RBS2_R and the region corresponding to *ibpfxs* with modified 5′-UTR amplified from pibpfxsT7lucA by using 014_pib_F and 014_pib_RBS2_R. The most optimal 5′-UTR sequence for *cfp* gene was chosen based on analysis of translation initiation rate predicted by RBS calculator [53]. In the case of *yfp* and *rfp*, the moderate strength SD8 RBSs and the stronger RBS from gene 10 of phage T7 were used, respectively, as proposed by Cox et al. [20]. Construction of pAG017 involved amplification of the *yfp* gene sequence together with TR2-17 and TL17 terminators from pZS2-123 by using YfpF_NdeI and YfpR_NotI. After digestion with NdeI and NotI of both the amplified pZS2-123 *cfp* and pAG001, the two fragments were ligated and the resulting vector was digested with MfeI and NdeI. The obtained backbone was ligated with the sequence of *rpsJ* promoter [54] synthesized as gBlock Gene Fragment (IDT) and digested with MfeI and NdeI prior to ligation. Construction of plasmid pAG032 was based on Gibson assembly of three fragments: the pAG002 backbone amplified using primers 032_002_F and 032_002_R, the *ibpfxs*::*cfp* region with RNAI terminator amplified from pAG014 using 032_014_F and 032_014_R and the *rpsJ*::*yfp* region with TR2-17 and TL17 terminators amplified from pAG017 using 032_017_F and 032_017_R. Additionally, two-gene versions of the reporter system were created. All these plasmids are available upon request. The *rpsJ*::*yfp* region with TR2-17 and TL17 terminators was amplified from pAG017 with primers rpsJ_yfpF_SnaBI and rpsJ_yfpR_NheI and cloned into pAG002 via SnaBI and NheI, generating pAG019. Similarly, SnaBI and NheI were used to clone the *ibpfxs*::*cfp* region with RNAI terminator amplified from pAG014 using pibfxs_cfpF_SnaBI and pibfxs_cfpR_NheI into pAG002. The ligation product was pAG024. Construction of plasmid pAG028 was based on Gibson assembly of two fragments: pAG017 backbone amplified with 021_rpsJ_F and 021_rpsJ_R and *ibpfxs*::*cfp* region with RNAI terminator amplified from pAG014 using 021_ibpfxs_F and 021_ibpfxs_R.

**Table 2:** Oligonucleotide primers used in this study

Name	Sequence (5′ → 3′)
a) PCR primers:	
RfpF_NdeI	ATATTCATATGGTTTCCAAGGGCGAGGAGG
RfpR_NotI	TTTATAGCGGCCGCAAAAACAGCCGTTGCCAGAAAG
014_cfp_F	ACCCTCCCTCGGCTTGTGCCGCGGCCGCAAAAGGAAAAGA
014_cfp_RBS2_R	GGAATATAGCAAATAAGGAGGAGGAAAATGAGCAAGGGCGAGGAGCTG
014_pib_F	AGCTTTTGTTCGGATCCAGCAAACTGCAAAAAAAAGTCCGCTGA
014_pib_RBS2_R	TTTCCTCCTCCTTATTTGCTATATTCCACCTGAATGGGTTGCGAATCGCGTTTAG
YfpF_NdeI	CGTGCATATGAGCAAAGGTGAAGAAC
YfpR_NotI	TGCTAGCGGCCGCAGGACAGCTATTGTAGATAAG
032_002_F	CCCGGTTTGATAGGGATAAG
032_002_R	TACGGATGAGCATTCATCAG
032_014_F	CTGATGAATGCTCATCCGTAGATCCAGCAAACTGCAAAAAAAAG
032_014_R	CAATAGCTGTCCTGCGGCCGATCGAGTTGCTGGAGATTGTG
032_017_F	CGGCCGCAGGACAGCTATTGTA
032_017_R	CTTATCCCTATCAAACCGGGATACCCTCCCTCGGCTTGTG
rpsJ_yfpF_SnaBI	GAATGCTCATCCGTAATTACCGCGAAACGGATACCCTCCCTC
rpsJ_yfpR_NheI	CTTATCCCTATCAAACCGGGGGCCTTTAGGTCTTCTTCTG
pibfxs_cfpF_SnaBI	CTTATCCCTATCAAACCGGGATCGAGTTGCTGGAGATTGT
pibfxs_cfpR_NheI	GAATGCTCATCCGTAATTACGGCCTTTAGGTCTTCTTCTG
021_rpsJ_F	AGCTTGACCTGTGAAGTGAA
021_rpsJ_R	CTGAGATCAGCTTTTGTTCG
021_ibpfxs_F	CGAACAAAAGCTGATCTCAGAGGCCTTTAGGTCTTCTTCT
021_ibpfxs_R	TTCACTTCACAGGTCAAGCTAAGGAAAAGATCCGGCAAAC
b) qPCR primers:	
cysG_F1	GTATTCCACTCACGCATCGC
cysG_R1	CGCCACCGGTTTTTAAGTGT
rplC_F2	TGACCGATAGAACCCGGAAC
rplC_R2	CTGGAACTTCCGTACCCAGG

### Expression studies and activity measurement of the reporters

Recombinant *E. coli*, *P. putida* and *A. vinelandii* strains were grown over-night in 2-10 ml of adequate liquid medium with ampicillin. Afterwards, 20 ml of the fresh medium with the antibiotic were inoculated with the overnight culture to an initial OD of 0.05 measured at 600 nm for *E. coli* and *P. putida* and at 660 nm for *A. vinelandii*. Following incubation in shake flasks, the XylS/*Pm*-mediated protein expression was induced in exponential phase (OD = 0.4–0.6) by adding m-toluic acid to a final concentration of 1 mM for *E. coli* and *P. putida* and 0.5 mM for *A. vinelandii*. Heat shock was applied by transferring the cultures of *P. putida* from 30 °C to 42 °C, 225 rpm shaking, at OD_600_ = 0.3–0.4. In order to reduce intracellular levels of ppGpp, 10 mM ammonium acetate was added to the cultures of *A. vinelandii* at OD_660_ = 0.3–0.4.

Fluorescence measurements were performed with the Tecan Infinite M200 (Tecan, Männedorf, Switzerland) plate reader. Fluorescence intensity was determined directly from the cultures (M63, Burk’s medium) or after resuspension in PBS (LB medium) in order to reduce background signal. The following fluorescent filter setup was used for the detection: [1] CFP excitation: 433 nm; emission: 475 nm; [2] YFP excitation: 505 nm; emission: 538 nm; [3] RFP excitation: 580 nm; emission: 615 nm. The gain was set to 100 for RFP and 80 for CFP and YFP.

### Transcript analysis by qPCR

Following adequate incubation in shake flasks, 3 ml of cell cultures were stabilized with RNAlater stabilization solution (Qiagen). The subsequent total RNA isolation was achieve using RNAqueous^®^ Total RNA Isolation Kit (Ambion). RNA samples were treated with TURBO™ DNase (Ambion) and used for cDNA synthesis with First-Strand cDNA Synthesis Kit (GE Healthcare Life Sciences). Two-step qPCR with the power PowerSYBR^®^ Green PCR Master Mix (Applied Biosystems) in QuantStudio™ 5 Real-Time PCR System (Applied Biosystems) was used for quantification of *cysG* and *rplC* transcripts. All steps were performed as described by the manufacturers. PCR cycles were 95 °C for 10 min, followed by 40 cycles of amplification (95 °C for 15 s; 62 °C for 1 min) and one additional stage of amplification to generate a melt curve. Results were analyzed with QuantStudio™ Design & Analysis Software (Applied Biosystems) and data were normalized as described previously [55]. Primer pairs used during amplification are listed in Table 2. Efficiency was verified for each pair of the primers (Table S2). Transcript generated from the *cysG* gene was used for normalization [56].

## Additional file

**Additional file 1: Table S1.** Growth rates (h^−1^) of *E. coli* BW25113 cells carrying pAG032. **Table S2.** Efficiencies of qPCR reaction (%) and R^2^ coefficients of determination of the standard curves for primer pairs used for amplification. **Figure S1.** Iinfluence of m-toluic acid (1 mM) on activity of (A) the XylS/*Pm* (only pAG032), (B) P*rspJ* and (C) P*ibpfxs* reporter units during cultivation of *E. coli* BW25113 transformed with three-gene version of the plasmid (pAG032) and two-gene version of the plasmid (pAG028). Cell were grown in M63 medium supplemented with glucose. The OD_600nm_ normalized fluorescence values were calculated from measurements taken at the time point corresponding to 3 h after the induction. The data presented are from three independent biological replica (average ± SD). Please note that values of OD normalized fluorescence, not relative fluorescence, are given.

## References

[1] Mahalik S, Sharma AK, Mukherjee KJ (2014). Genome engineering for improved recombinant protein expression in *Escherichia coli*. Microb Cell Fact.

[2] Rogers JK, Taylor ND, Church GM (2016). Biosensor-based engineering of biosynthetic pathways. Curr Opin Biotechnol.

[3] Chou CP (2007). Engineering cell physiology to enhance recombinant protein production in *Escherichia coli*. Appl Microbiol Biotechnol.

[4] Lemke JJ, Sanchez-Vazquez P, Burgos HL, Hedberg G, Ross W, Gourse RL (2011). Direct regulation of *Escherichia coli* ribosomal protein promoters by the transcription factors ppGpp and DksA. Proc Natl Acad Sci USA..

[5] Liu K, Bittner AN, Wang JD (2015). Diversity in (p)ppGpp metabolism and effectors. Curr Opin Microbiol.

[6] Gaca AO, Kajfasz JK, Miller JH, Liu K, Wang JD, Abranches J (2013). Basal levels of (p)ppGpp in *Enterococcus faecalis*: the magic beyond the stringent response. MBio..

[7] Potrykus K, Cashel M (2008). (p)ppGpp: still magical?. Annu Rev Microbiol.

[8] Ross W, Vrentas CE, Sanchez-Vazquez P, Gaal T, Gourse RL (2013). The magic spot: a ppGpp binding site on *E. coli* RNA polymerase responsible for regulation of transcription initiation. Mol Cell..

[9] Hoffmann F, Rinas U (2004). Roles of heat-shock chaperones in the production of recombinant proteins in *Escherichia coli*. Adv Biochem Eng Biotechnol.

[10] Neubauer P, Fahnert B, Lilie H, Villaverde A, Shively JM (2006). Protein inclusion bodies in recombinant bacteria. Inclusions in prokaryotes.

[11] Kraft M, Knupfer U, Wenderoth R, Pietschmann P, Hock B, Horn U (2007). An online monitoring system based on a synthetic sigma32-dependent tandem promoter for visualization of insoluble proteins in the cytoplasm of *Escherichia coli*. Appl Microbiol Biotechnol.

[12] Nakahigashi K, Yanagi H, Yura T (1995). Isolation and sequence analysis of rpoH genes encoding sigma 32 homologs from Gram negative bacteria: conserved mRNA and protein segments for heat shock regulation. Nucleic Acids Res.

[13] Nakahigashi K, Yanagi H, Yura T (1998). Regulatory conservation and divergence of sigma32 homologs from Gram-negative bacteria: serratia marcescens, *Proteus mirabilis*, *Pseudomonas aeruginosa*, and *Agrobacterium tumefaciens*. J Bacteriol.

[14] Kitagawa M, Matsumura Y, Tsuchido T (2000). Small heat shock proteins, IbpA and IbpB, are involved in resistances to heat and superoxide stresses in *Escherichia coli*. FEMS Microbiol Lett.

[15] Lesley SA, Graziano J, Cho CY, Knuth MW, Klock HE (2002). Gene expression response to misfolded protein as a screen for soluble recombinant protein. Protein Eng.

[16] Siurkus J, Panula-Perala J, Horn U, Kraft M, Rimseliene R, Neubauer P (2010). Novel approach of high cell density recombinant bioprocess development: optimisation and scale-up from microliter to pilot scales while maintaining the fed-batch cultivation mode of *E. coli* cultures. Microb Cell Fact..

[17] Nikolay R, Schloemer R, Schmidt S, Mueller S, Heubach A, Deuerling E (2014). Validation of a fluorescence-based screening concept to identify ribosome assembly defects in *Escherichia coli*. Nucleic Acids Res.

[18] Failmezger J, Ludwig J, Niess A, Siemann-Herzberg M (2017). Quantifying ribosome dynamics in *Escherichia coli* using fluorescence. FEMS Microbiol Lett..

[19] Blatny JM, Brautaset T, Winther-Larsen HC, Haugan K, Valla S (1997). Construction and use of a versatile set of broad-host-range cloning and expression vectors based on the RK2 replicon. Appl Environ Microbiol.

[20] Cox RS, Dunlop MJ, Elowitz MB (2010). A synthetic three-color scaffold for monitoring genetic regulation and noise. J Biol Eng..

[21] Gawin A, Valla S, Brautaset T (2017). The XylS/Pm regulator/promoter system and its use in fundamental studies of bacterial gene expression, recombinant protein production and metabolic engineering. Microb Biotechnol.

[22] Burgos HL, O'Connor K, Sanchez-Vazquez P, Gourse RL (2017). Roles of transcriptional and translational control mechanisms in regulation of ribosomal protein synthesisin *Escherichia coli*. J Bacteriol..

[23] Navarro Llorens JM, Tormo A, Martinez-Garcia E (2010). Stationary phase in Gram-negative bacteria. FEMS Microbiol Rev.

[24] Singha TK, Gulati P, Mohanty A, Khasa YP, Kapoor RK, Kumar S (2017). Efficient genetic approaches for improvement of plasmid based expression of recombinant protein in *Escherichia coli*: a review. Process Biochem.

[25] Blatny JM, Brautaset T, Winther-Larsen HC, Karunakaran P, Valla S (1997). Improved broad-host-range RK2 vectors useful for high and low regulated gene expression levels in Gram-negative bacteria. Plasmid.

[26] Figurski DH, Meyer RJ, Helinski DR (1979). Suppression of Co1E1 replication properties by the Inc P-1 plasmid RK2 in hybrid plasmids constructed in vitro. J Mol Biol.

[27] Sletta H, Nedal A, Aune TE, Hellebust H, Hakvag S, Aune R (2004). Broad-host-range plasmid pJB658 can be used for industrial-level production of a secreted host-toxic single-chain antibody fragment in *Escherichia coli*. Appl Environ Microbiol.

[28] Klumpp S, Scott M, Pedersen S, Hwa T (2013). Molecular crowding limits translation and cell growth. Proc Natl Acad Sci USA..

[29] Scott M, Gunderson CW, Mateescu EM, Zhang Z, Hwa T (2010). Interdependence of cell growth and gene expression: origins and consequences. Science.

[30] Dennis PP (1976). Effects of chloramphenicol on the transcriptional activities of ribosomal RNA and ribosomal protein genes in *Escherichia coli*. J Mol Biol.

[31] Huang HC, Sherman MY, Kandror O, Goldberg AL (2001). The molecular chaperone DnaJ is required for the degradation of a soluble abnormal protein in *Escherichia coli*. J Biol Chem.

[32] Kanemori M, Mori H, Yura T (1994). Induction of heat shock proteins by abnormal proteins results from stabilization and not increased synthesis of sigma 32 in *Escherichia coli*. J Bacteriol.

[33] Prouty WF, Karnovsky MJ, Goldberg AL (1975). Degradation of abnormal proteins in *Escherichia coli*. Formation of protein inclusions in cells exposed to amino acid analogs. J Biol Chem.

[34] Li Z, Nimtz M, Rinas U (2017). Global proteome response of *Escherichia coli* BL21 to production of human basic fibroblast growth factor in complex and defined medium. Eng Life Sci.

[35] Valgepea K, Adamberg K, Seiman A, Vilu R (2013). *Escherichia coli* achieves faster growth by increasing catalytic and translation rates of proteins. Mol BioSyst.

[36] Sletta H, Tondervik A, Hakvag S, Aune TE, Nedal A, Aune R (2007). The presence of N-terminal secretion signal sequences leads to strong stimulation of the total expression levels of three tested medically important proteins during high-cell-density cultivations of *Escherichia coli*. Appl Environ Microbiol.

[37] Setubal JC, dos Santos P, Goldman BS, Ertesvag H, Espin G, Rubio LM (2009). Genome sequence of *Azotobacter vinelandii*, an obligate aerobe specialized to support diverse anaerobic metabolic processes. J Bacteriol.

[38] Lieder S, Nikel PI, de Lorenzo V, Takors R (2015). Genome reduction boosts heterologous gene expression in *Pseudomonas putida*. Microb Cell Fact.

[39] Ito F, Tamiya T, Ohtsu I, Fujimura M, Fukumori F (2014). Genetic and phenotypic characterization of the heat shock response in *Pseudomonas putida*. Microbiologyopen..

[40] Jakociunas T, Jensen MK, Keasling JD (2016). CRISPR/Cas9 advances engineering of microbial cell factories. Metab Eng.

[41] Mougiakos I, Bosma EF, Ganguly J, van der Oost J, van Kranenburg R (2018). Hijacking CRISPR-Cas for high-throughput bacterial metabolic engineering: advances and prospects. Curr Opin Biotechnol.

[42] Bosdriesz E, Molenaar D, Teusink B, Bruggeman FJ (2015). How fast-growing bacteria robustly tune their ribosome concentration to approximate growth-rate maximization. FEBS J.

[43] Klumpp S, Zhang Z, Hwa T (2009). Growth rate-dependent global effects on gene expression in bacteria. Cell.

[44] Balzer S, Kucharova V, Megerle J, Lale R, Brautaset T, Valla S (2013). A comparative analysis of the properties of regulated promoter systems commonly used for recombinant gene expression in *Escherichia coli*. Microb Cell Fact.

[45] Farr SB, Kogoma T (1991). Oxidative stress responses in *Escherichia coli* and *Salmonella typhimurium*. Microbiol Rev.

[46] VanBogelen RA, Kelley PM, Neidhardt FC (1987). Differential induction of heat shock, SOS, and oxidation stress regulons and accumulation of nucleotides in *Escherichia coli*. J Bacteriol.

[47] Cardinale S, Joachimiak MP, Arkin AP (2013). Effects of genetic variation on the *E. coli* host-circuit interface. Cell Rep..

[48] Datsenko KA, Wanner BL (2000). One-step inactivation of chromosomal genes in *Escherichia coli* K-12 using PCR products. Proc Natl Acad Sci USA..

[49] Ramos-Gonzalez MI, Campos MJ, Ramos JL (2005). Analysis of *Pseudomonas putida* KT2440 gene expression in the maize rhizosphere: in vivo [corrected] expression technology capture and identification of root-activated promoters. J Bacteriol.

[50] Elbing K, Brent R (2002). Media preparation and bacteriological tools. Curr Protoc Mol Biol..

[51] Hoidal HK, Glaerum Svanem BI, Gimmestad M, Valla S (2000). Mannuronan C-5 epimerases and cellular differentiation of *Azotobacter vinelandii*. Environ Microbiol.

[52] Gibson DG, Young L, Chuang RY, Venter JC, Hutchison CA, Smith HO (2009). Enzymatic assembly of DNA molecules up to several hundred kilobases. Nat Methods.

[53] Salis HM, Mirsky EA, Voigt CA (2009). Automated design of synthetic ribosome binding sites to control protein expression. Nat Biotechnol.

[54] Zurawski G, Zurawski SM (1985). Structure of the *Escherichia coli* S10 ribosomal protein operon. Nucleic Acids Res.

[55] Livak KJ, Schmittgen TD (2001). Analysis of relative gene expression data using real-time quantitative PCR and the 2(−Delta Delta C(T)) Method. Methods.

[56] Zhou K, Zhou L, Lim Q, Zou R, Stephanopoulos G, Too HP (2011). Novel reference genes for quantifying transcriptional responses of *Escherichia coli* to protein overexpression by quantitative PCR. BMC Mol Biol.

