# Supplementary material for: Construction and characterization of broad-host-range reporter plasmid suitable for on-line analysis of bacterial host responses related to recombinant protein production
Source: Microb Cell Fact. 2019 May 7;18:80. doi: 10.1186/s12934-019-1128-7 (PMC6505264; doi:10.1186/s12934-019-1128-7)

**ADDITIONAL MATERIAL**

Table S1. Growth rates (h^−1^) of *E. coli* BW25113 cells carrying pAG032.

| **Conditions** | **Medium composition** | | |
| --- | --- | --- | --- |
|  | M63/glycerol | M63/glucose | M63/glucose + CAA |
| non-induced | 0.388 | 0.523 | 0.574 |
| induced | 0.352 | 0.501 | 0.469 |

Recombinant cells were cultivated under non-induced and induced conditions in M63 minimal medium with different supplementation. The growth rates were calculated from OD_600nm_ values measured every 1 h from 0 to 3 h from induction with the m-toluic acid. The data presented are from three independent biological replica (average±SD).

Table S2. Efficiencies of qPCR reaction (%) and R^2^ coefficients of determination of the standard curves for primer pairs used for amplification.

| **Gene name** | **Efficiency** | **R^2^** |
| --- | --- | --- |
| *cysG* | 95.91 | 0.999 |
| *rplC* | 98.52 | 0.999 |

Figure S1. Iinfluence of m-toluic acid (1 mM) on activity of (A) the XylS/*Pm* (only pAG032), (B) P*rspJ* and (C) P*ibpfxs* reporter units during cultivation of *E. coli* BW25113 transformed with three-gene version of the plasmid (pAG032) and two-gene version of the plasmid (pAG028). Cell were grown in M63 medium supplemented with glucose. The OD_600nm_ normalized fluorescence values were calculated from measurements taken at the time point corresponding to 3 h after the induction. The data presented are from three independent biological replica (average±SD). Please note that values of OD normalized fluorescence, not relative fluorescence, are given.


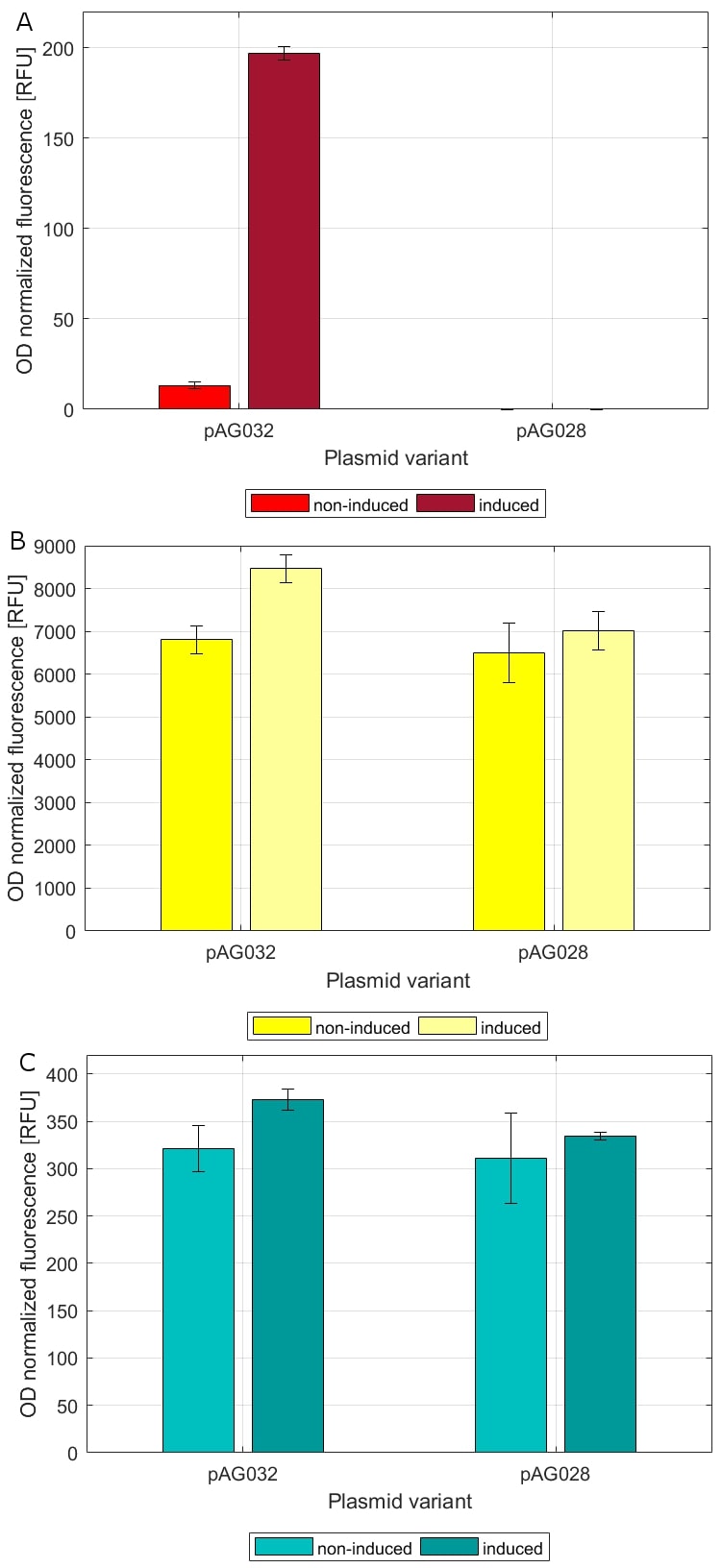

Supplement: Supplementary file 1 — Additional file 1: Table S1. Growth rates (h−1) of E. coli BW25113 cells carrying pAG032. Table S2. Efficiencies of qPCR reaction (%) and R2 coefficients of determination of the standard curves for primer pairs used for amplification. Figure S1. Iinfluence of m-toluic acid (1 mM) on activity of (A) the XylS/Pm (only pAG032), (B) PrspJ and (C) Pibpfxs reporter units during cultivation of E. coli BW25113 transformed with three-gene version of the plasmid (pAG032) and two-gene version of the plasmid (pAG028). Cell were grown in M63 medium supplemented with glucose. The OD600nm normalized fluorescence values were calculated from measurements taken at the time point corresponding to 3 h after the induction. The data presented are from three independent biological replica (average ± SD). Please note that values of OD normalized fluorescence, not relative fluorescence, are given. [file 12934_2019_1128_MOESM1_ESM.docx]
